# Interleukin‐25 initiates Th2 differentiation of human CD4^+^ T cells and influences expression of its own receptor

**DOI:** 10.1002/iid3.87

**Published:** 2015-10-15

**Authors:** Graeme Bredo, Jessica Storie, Nami Shrestha Palikhe, Courtney Davidson, Alexis Adams, Harissios Vliagoftis, Lisa Cameron

**Affiliations:** ^1^ Pulmonary Research Group, Department of MedicineUniversity of AlbertaEdmontonAlbertaCanada; ^2^ Department of Pathology and Laboratory Medicine, Schulich School of Medicine & DentistryWestern UniversityLondonOntarioCanada

**Keywords:** CRTh2, GATA3, IL‐4, IL‐25, IL‐25 receptor, Th2 differentiation

## Abstract

Human CRTh2^+^ Th2 cells express IL‐25 receptor (IL‐25R) and IL‐25 has been shown to potentiate production of Th2 cytokines. However, regulation of IL‐25R and whether it participates in Th2 differentiation of human cells have not been examined. We sought to characterize IL‐25R expression on CD4^+^ T cells and determine whether IL‐25 plays a role in Th2 differentiation. Naïve human CD4^+^ T cells were activated in the presence of IL‐25, IL‐4 (Th2 conditions) or both cytokines to assess their relative influence on Th2 differentiation. For experiments with differentiated Th2 cells, CRTh2‐expressing cells were isolated from differentiating cultures. IL‐25R, GATA3, CRTh2 and Th2 cytokine expression were assessed by flow cytometry, qRT‐PCR and ELISA. Expression of surface IL‐25R was induced early during Th2 differentiation (2 days). Addition of IL‐25 to naïve CD4^+^ T cells revealed that it induces expression of its own receptor, more strongly than IL‐4. IL‐25 also increased the proportions of IL‐4‐, GATA3‐ and CRTh2‐expressing cells and expression of IL‐5 and IL‐13. Activation of differentiated CRTh2^+^ Th2 cells through the TCR or by CRTh2 agonist increased surface expression of IL‐25R, though re‐expression of CRTh2 following TCR downregulation was impeded by IL‐25. These data suggest that IL‐25 may play various roles in Th2 mediated immunity. We establish here it regulates expression of its own receptor and can initiate Th2 differentiation, though not as strongly as IL‐4.

## Introduction

TSLP, IL‐33 and IL‐25, produced by airway epithelium in response to a wide variety of environmental stimuli, are now considered important links between innate immune responses and development of Th2 immunity (reviewed in 1). TSLP‐activated dendritic cells promote Th2 cell infiltration to the airways 2 and maintenance of the Th2 phenotype through the OX40‐OX40L pathway 3. IL‐33 and IL‐25 support dendritic cells in mediating Th2 cell responses 4, 5 and have also been shown to drive the group 2 innate lymphoid cells (ILC2) to produce IL‐4, IL‐5 and IL‐13 6, 7, 8. However, whether these epithelial cytokines can initiate Th2 differentiation is not clear. Hurst et. al. showed that the production of IL‐5 and IL‐13, eosinophilia, mucous production and airway hyper‐responsiveness (AHR) induced by administration of IL‐25 to the airways was independent of T cells and IL‐4 9, while Sharkhuu and colleagues reported that IL‐25 induced IL‐4 as well as IL‐5 and IL‐13 from naïve murine T cells 10. Since, RSV‐induced Th2 inflammation and airway responses were severely impaired in animals deficient in IL‐25 receptor (R) 11, IL‐25 may be a link between viral infections and development of asthma. In humans, expression of IL‐25 and its receptor are higher in bronchial biopsies from asthmatics than normal controls and skin from patients with atopic dermatitis compared to control skin 5, 12. As such, studying how IL‐25 influences human Th2 responses is an important step toward understanding the role of IL‐25‐inducing environmental stimuli in the development of allergic disease.

IL‐4 is considered imperative for driving Th2 differentiation and which cell types provide ‘early’ IL‐4 has been a longstanding question 13, 14, 15, 16, 17. However, the necessity of paracrine sources of IL‐4 is controversial 18, as various groups have shown that T cells can produce enough IL‐4 to support Th2 differentiation (reviewed in 19). Indeed, initiation of Th2 differentiation commences when naïve CD4^+^ T cells are activated by antigen induced T cell receptor (TCR) crosslinking. This results in low level IL‐4 expression mediated by transcription factors such as NFAT/AP‐1 20 and NF‐κB 21. During the reinforcement stage IL‐4R and TCR signaling, through STAT6 and NFκB, work together to upregulate GATA3 22, 23, which significantly increases IL‐4 production 24. Other transcription factors, such as cMAF, JunB and STAT5 also enhance IL‐4 expression during differentiation 25, 26, 27. Maintenance of the Th2 phenotype is mediated by chromatin remodelling of Th2 loci resulting in differential accessibility of regulatory regions, ultimately releasing Th2 cells from their dependence on continued stimulation with IL‐4 (reviewed in 28). These cells express CRTh2, which has been shown to mark the differentiated Th2 phenotype 29. Though relatively little is known regarding regulation of CRTh2, GATA3 can induce its expression on naïve human T cells 30 and also activate the CRTh2 promoter 31. As such, mediators acting on naïve CD4^+^ T cells to enhance expression of IL‐4 or other Th2 factors may influence Th2 lineage commitment and CRTh2 expression.

CRTh2 is a receptor for PGD_2_ 32, 33, a lipid mediator released by activated mast cells (reviewed in 34). PGD_2_ activation of CRTh2^+^ Th2 cells mediates chemotaxis and influx of CRTh2^+^ cells into the tissue 35 and also induces expression of the Th2 cytokines 36. CRTh2^+^ Th2 cells are CD62L^+^ memory cells that circulate between the periphery and lymph nodes 3. Activation through CRTh2 has been shown to mediate movement of memory CD4^+^ T cells through lymphatic vascular endothelial cells 37, an *in vitro* model of T cell egress from the periphery. Therefore, the PGD_2_‐CRTh2 pathway is considered to potentiate infiltration of cells to inflammatory sites as well as exit during the resolution phase.

Stimulation of naïve murine CD4^+^ T cells with IL‐25 has been shown to induce IL‐4 and GATA3 38, indicating it may play a role in Th2 differentiation. However, numerous species differences have been observed between the murine and human immune system. For instance, the importance of certain molecules for TCR signaling, mechanisms regulating intracellular calcium, leukocyte transit times and the role of various chemokine families appear to differ between humans and mice (reviewed in 39). In the case of Th2 cells, CRTh2 is considered a marker of human 40, but not mouse Th2 cells 41 and De Fanis *et al*. have shown that in humans only the CRTh2^+^ Th2 cell population exhibits levels of GATA3 significantly higher than Th1 cells 42. Collectively, these studies highlight the importance of specifically examining Th2 differentiation of human cells. Regulation of IL‐25R expression and characterization of when it appears during Th2 differentiation have not been studied. Though IL‐25 has been shown to enhance Th2 cytokine expression from memory Th2 cells, particularly in the presence of dendritic cell co‐culture 5, whether IL‐25 plays a role in Th2 differentiation of human T cells is not known.

In this study, we sought to determine the effect of IL‐25 on human CD4^+^ T cells. We assessed its influence on expression of the IL‐25R, Th2 differentiation and ability to induce soluble mediator expression. We demonstrate that IL‐25 is able to act directly on naïve CD4^+^ T cells to initiate Th2 differentiation, though not as strongly as IL‐4. Furthermore, on differentiated Th2 cells IL‐25 lowered CRTh2 expression, indicating it may influence Th2 cell emigration out of tissues and resolution of inflammation.

## Materials and Methods

### Th2 differentiation

This study was approved by the University of Alberta Human Ethics Review Board (approval number 00000942). Human blood was collected from self‐reported non‐allergic, non‐asthmatic volunteers using sodium heparin tubes. Dilute blood (1:2 PBS) was layered over Ficoll (Histopaque PLUS, GE Healthcare, Upsala, Sweden) and separated by density centrifugation (30 min, room temperature (RT), 2200 RPM) with no break. Peripheral blood mononuclear cells (PBMC) were collected from the buffy coat and naïve CD4^+^ T cells isolated by negative selection according to manufacturer's instructions (Cat. #130‐094‐131, Miltenyi Biotech, Auburn, CA) resulting in highly pure populations (CD3, >99%; CD4, 97%; CD8, <1%; CD14, <1%; CD45RA, 93–95%). Cells were cultured (2 × 10^6^ cells/mL) in X‐vivo 15 media (Lonza, Cat. #04‐744Q, Walkersville, MD) supplemented with penicillin (100 U/mL), streptomycin (100 mg/mL), L‐glutamine (292 μg/mL; (Cat. #10378‐016, Gibco, Waltman, MA) and 10% HyClone fetal bovine serum (Cat. #SH30070.03, Thermo Scientific, Waltman, MA). Cells were activated (3 days) on plates coated with antibody (α) against CD3 (αCD3, 1 µg/mL) and αCD28 (1 µg/mL) in non‐polarizing control conditions (rhIL‐2 [2.5 ng/mL], αIFNγ [1 µg/mL] and αIL‐12 [1 µg/mL]), Th2 conditions (rhIL‐4 [50 ng/mL], rhIL‐2, αIFNγ and αIL‐12) and both these conditions in the presence of rhIL‐25 (50 ng/mL). Cells were taken off αCD3/αCD28, replenished with fresh cytokine/antibody combinations and expanded (4 days). Cells were carried on subsequent cycles of activation and proliferation for up to 38 days. To assess the effect of IL‐25, independent of IL‐4, some experiments were carried out with these conditions in addition to blocking antibody against IL‐4 (2 μg/mL). Reagents were purchased from R&D Systems (Minneapolis, MN, USA: αCD3, Cat. #MAB100 (clone UCHT‐1); αCD28 (clone 37407), Cat. #MAB342; rhIL‐2, Cat. #202‐IL‐010; rhIL‐4, Cat. #204‐IL‐010; αIFNγ, Cat. #AF‐285‐NA; rhIL‐25, Cat. #1258‐IL‐025; anti‐IL‐4, Cat. #MAD204), while anti‐IL‐12 (Cat. #16‐7129) was from eBiosciences (San Diego, CA, USA). Purity of recombinant proteins was >97%, by SDS‐Page and endotoxin levels were < 0.10 EU/1 μg of protein by the LAL method.

### Differentiated CRTh2^+^ Th2 cell lines

To generate a CRTh2 enriched Th2 cell line, the above Th2 differentiation protocol was followed. On day 14, αCRTh2 antibody coated microbeads (Cat. #130‐091‐274, Miltenyi) were used to isolate CRTh2‐expressing cells. CRTh2^+^ Th2 cells were then cultured on cycles of activation (3 days, αCD3/αCD28 and rhIL‐2) and proliferation (4 days, rhIL‐2) for up to 49 days.

### Quantitative reverse transcription (qRT‐PCR)

To perform qRT‐PCR, RNA was extracted using RNAeasy extraction kit (Cat. #74101, ON, Canada) and eluted with 30 µl of RNase/DNase free water. Complementary DNA (cDNA) was synthesized from 1 µg of RNA using the Superscript II Reverse Transcriptase according to manufacturer's instructions (Cat #18964‐014, Invitrogen, Burlington, ON, Canada). qRT‐PCR TaqMan gene expression assays for CRTh2 (Hs00173717_m1), IL‐25R (Hs00218889_1) and IL‐4 (Hs00174122_m1) were purchased from Applied Biosystems (Burlington, On, Canada). The PCR program was 2 min at 50°C, 10 min at 95°C, 40 cycles of 15 sec at 95°C, 1 min at 60°C (Eppendorf RealPlex 4, Mississauga, ON). Data were analyzed using the ΔΔCycle threshold (Ct) compared to GAPDH method. Amplification of GAPDH was performed using a custom 6FAM‐labeled TAMRA probe (5′‐AAA TCC CAT CAC CAT CTT CCA GGA GCG A‐3′; Applied Biosystems) with a forward primer primer (5′‐CTG AGA ACG GGA AGC TTG TCA‐3′) and reverse primer (5′‐GCA AAT GAG CCC CAG CCT T‐3′). Briefly ΔCt was determined by subtracting the Ct of the housekeeping gene from the Ct of the test gene. The ΔCt from the control condition was subtracted from experimental condition to determine the ΔΔCt. The fold increase was then calculated by using the ΔΔCt as a negative exponent to the base of 2 (2^−ΔΔCt^).

### Flow cytometry

Phenotypic characterization of both differentiating Th2 cells and CRTh2‐isolated Th2 cells was carried out by flow cytometry, after proliferation. Cells were blocked (30 min) with normal rat IgG (Invitrogen, Cat# 10700, Burlington, ON) followed by incubation (30 min, 4°C) with either the isotype matched control or marker specific antibody. Finally, cells were placed into paraformaldehyde (2%)/sucrose (0.54%). Cells were stained for CD4 (Clone RPA‐T4, mouse IgG1 FITC, AbD Serotech, Raleigh, NC) and CD45RA (Clone L48, mouse IgG1κ FITC, BD Pharmingen, Mississauga, ON). Biotinylated antibodies were used to assess surface CRTh2 and IL‐25R. For CRTh2, cells were blocked (30 min, room temperature) with rat IgG (Invitrogen, Burlington, ON) then stained with primary biotin conjugated αCRTh2 antibody (Clone BM16, Miltenyi) or rat IgG2a isotype (AbD serotech, Raleigh, NC) (30 min, 4°C). Incubation with streptavidin‐APC (30 min, 4°C) (eBioscience, San Diego, CA) was used as detection. Staining for IL‐25R followed the same protocol but blocking was with goat IgG then stained with primary biotin‐conjugated αIL‐17RB (Cat. #BAF1207, R&D Systems, Minneapolis, MN) or goat anti‐human IgG isotype (BAF108, R&D Systems, Minneapolis, MN) followed by incubation with streptavidin‐APC (30 min, 4°C). Intracellular staining for cytokines was performed after 4 h stimulation with PMA (20 ng/mL), ionomycin (1 µM) and brefaldin A (10 µg/mL), while assessment of intracellular GATA3 was on unstimulated cells. Cells were fixed (10 min, on ice) with paraformaldehyde (4%; Sigma Aldrich, Oakville, ON) and permeabilized (10 min, on ice) with saponin (0.4%; Sigma Aldrich, Oakville, ON). For non‐permeabilized controls saponin was replaced with PBS. Antibodies and isotype controls were added and samples were incubated (30 min, on ice). Cells were stained with IL‐4‐Alexa‐488 (Clone 8D4‐8), IL‐13‐PE (Clone JES10‐5A2), IFNγ‐Alexa 647 (Clone B27) and GATA3‐Alexa‐488 (Clone L50‐823). Data were acquired immediately after staining using either FACSCalibur or LSRII (Becton Dickson, Mississauga, ON). Results were analyzed using FlowJo (Tree Star, Ashland, OR). Positive signal was determined by setting the gates on the isotype control and expression obtained by antibody shift.

### Measurement of Th2 cytokine production

Supernatants were examined for cytokine production after αCD3/αCD28 activation. For experiments from differentiating Th2 cells, supernatants (days 3 and 10) were examined for cytokine expression using a laser multiplex bead array (Eve Technologies, Calgary, AB: limit of detection: 0.64 pg/mL). For experiments examining the longterm effect of adding IL‐25 to Th2 conditions, ELISA for IL‐5 (Cat. #S5000B, R&D Systems, Minneapolis, MN, limit of detection: 3.9 pg/mL) and IL‐13 (Cat. #851 630 005, Diaclone Besancon Cedex, France, limit of detection: 1.5 pg/mL) were performed as described by the manufacturer. Briefly, plates were coated with capture antibody (4°C, overnight), samples were blocked with isotype (2 h, RT) and then loaded into wells and incubated (2 h, RT). Enzyme‐conjugated antibodies were added and quantity was assayed by color change following substrate addition. Samples were tested in duplicate. ELISA plates were read in a Powerwave XS Microplate Reader (Bio‐Tek, Winooski, VT).

### Statistics

All data are expressed as the mean ± standard error of the mean (SEM), except for some IL‐5 time points (*n* = 2), where standard deviation (SD) was used. Differences between conditions were determined by paired *t* test, repeated measures ANOVA with Dunnett's or one‐way ANOVA with Tukey's method for multiple comparison. Significance was assumed at *P* < 0.05. All analyses were conducted with SigmaPlot (v12.3, Systat Software, Inc., San Jose, CA, USA).

## Results

### IL‐25R is expressed by CRTh2^+^ Th2 cells differentiated *in vitro*

**Figure 1:**
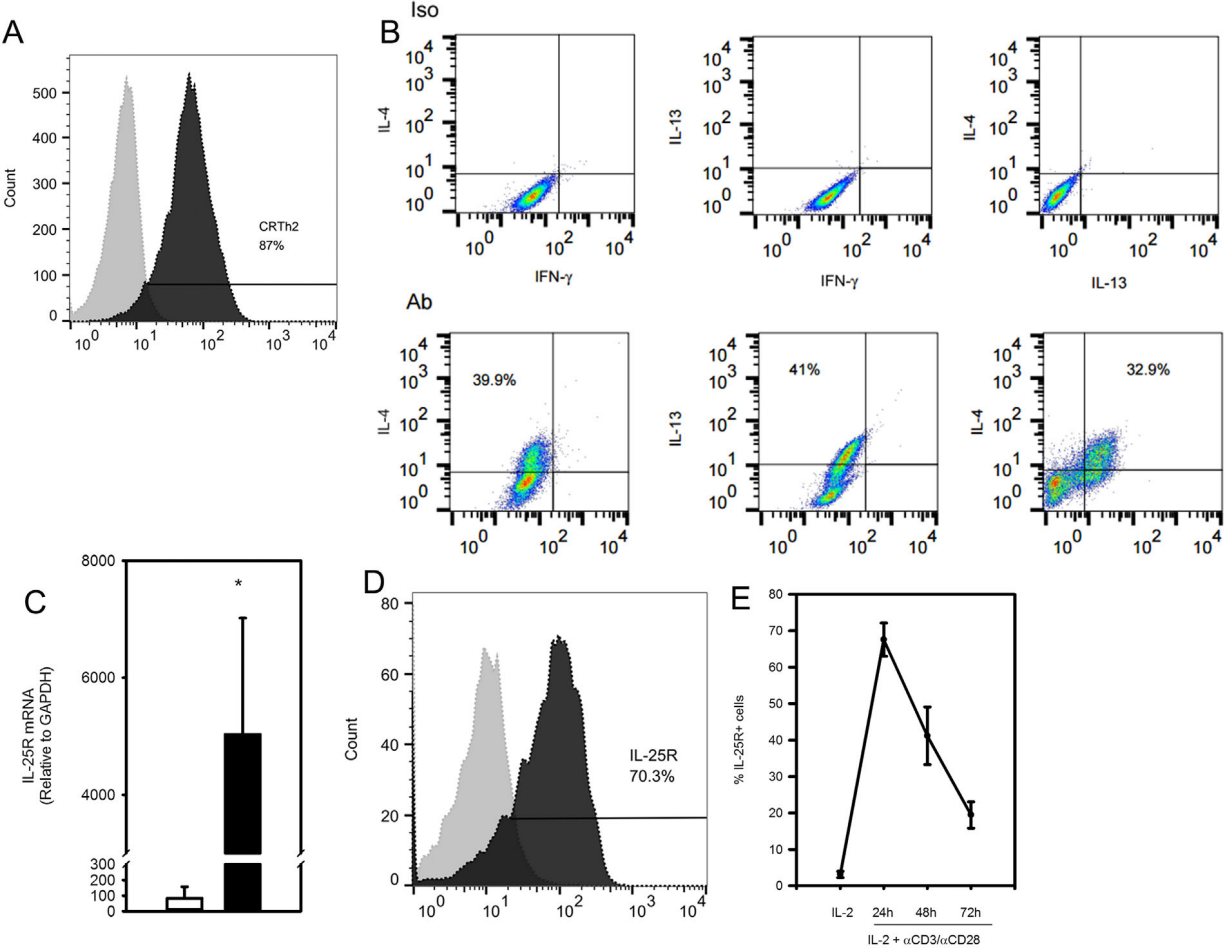
IL‐25R is highly expressed by *in vitro* differentiated CRTh2^+^ Th2 cells. Flow cytometry of (A) surface CRTh2 (solid line) compared with isotype control (dotted line; day 42) and (B) intra‐cellular IL‐4, IL‐13 and IFN‐γ⋅ (C) qRT‐PCR for IL‐25R mRNA from activated CRTh2^+^ Th2 cells (day 45, black bar, *n* = 6 independently differentiated lines) were compared with activated non‐polarized CD4**^+^** T cells (day 3, white bar, 61‐fold, *n* = 5). (D) Representative example of surface IL‐25R expression (solid line) compared to isotype control (dotted line) by differentiated CRTh2**^+^** Th2 cells (day 43) and (E) kinetics of surface IL‐25R expression following TCR activation (*n* = 4, three independently differentiated lines). Statistical significance was determined by Student's *t* test, *P < 0.05.

Human memory CRTh2^+^ Th2 cells isolated from peripheral blood have been shown to express the IL‐25R 5. To investigate whether *in vitro* differentiated cells also express this receptor, naïve CD4^+^ T cells were cultured in the presence of Th2 polarizing conditions, the CRTh2‐expressing cells isolated (day 14) and then maintained on weekly cycles of activation (3 days) and rest (4 days) in the presence of IL‐2. This protocol generated Th2 cell lines highly positive for CRTh2 (day 42; Fig. 1A) and showed a Th2 polarized cytokine profile (Fig. 1B). These cells also exhibited a significantly higher level of IL‐25R mRNA than non‐polarized CD4^+^ T cells (Fig. 1C, 61‐fold; *P* < 0.05) and robust surface expression IL‐25R (Fig. 1D). Kinetics of IL‐25R expression was also assessed and showed that expression of IL‐25R was low after resting conditions and induced by activation, with maximum expression after 24 h (Fig. 1E).

### IL‐25R is expressed early during Th2 differentiation

**Figure 2:**
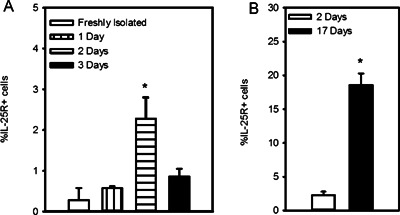
IL‐25R is expressed early in Th2 differentiation. (A) Flow cytometry of surface IL‐25R on naïve CD4**^+^** T cells freshly isolated from peripheral blood after 1, 2, or 3 days in Th2 conditions. (B) IL‐25R expression early (day 2) and later (day 17) during differentiation were compared (*n* = 3 independently differentiated lines). Statistical significance determined by ANOVA (A) or Student's *t* test (B), *P < 0.05.

To further characterize IL‐25R, we next examined the kinetics of its expression by naïve CD4^+^ T cells undergoing Th2 differentiation. Figure 2A shows that surface IL‐25R was not expressed by freshly isolated naïve CD4^+^ T cells (0.30 ± 0.3%), but a low level was observed as early as 2 days of Th2 differentiating conditions (2.3 ± 0.5%). This expression was substantially upregulated by day 17 (18.6 ± 1.7%), which was after 3 days of activation during a 3rd round of polarization (Fig. 2B).

### IL‐25 initiates acquisition of the Th2 phenotype

**Figure 3:**
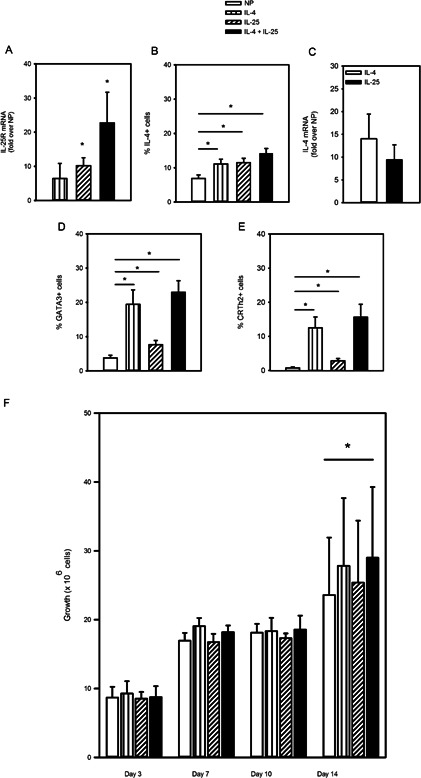
IL‐25 mediates acquisition of the Th2 phenotype. Naïve CD4**^+^** T cells were differentiated in non‐polarizing (NP) control conditions (αCD3/αCD28, IL‐2, αIFNγ and αIL‐12) or NP + IL‐4 (IL‐4), NP + IL‐25 (IL‐25) or NP + IL‐4 and IL‐25 (IL‐4+IL‐25). (A) IL‐25R mRNA expression (fold increase over NP; day 3, 7, 10, 14, *n* = 10 of three independently differentiated lines). (B) Intracellular IL‐4 and (E) surface CRTh2 were assayed following proliferation (day 7, 14, *n* = 17, 10 independently differentiated lines). (D) GATA3 was assayed after stimulation (day 3, 10, *n* = 17, 10 independently differentiated lines). (C) IL‐4 mRNA expression by cells cultured in IL‐4 or IL‐25 (fold increase over NP, day 14; *n* = 4 independently differentiated lines). (F) Cell counts were determined and comparison was made between the four conditions for each day. Statistical significance determined by repeated measures ANOVA, *P < 0.05.

Since we observed that IL‐25R is expressed early following exposure to Th2 conditions, we asked whether IL‐25 could influence Th2 differentiation. To examine this, cells were cultured in non‐polarizing (NP) control conditions (IL‐2, αIFNγ, αIL‐12) or NP along with IL‐4, IL‐25 or both cytokines to test their relative and synergistic capacity for Th2 differentiation. Therefore, data are reported as (*i*) NP, (*ii*) IL‐4 (NP + IL‐4, typical Th2 conditions), (*iii*) IL‐25 (NP + IL‐25) and (*iv*) IL‐4 + IL‐25 (NP + IL‐4 and IL‐25). IL‐25 induced expression of IL‐25R mRNA (10‐fold over NP), significantly more than IL‐4 (6‐fold, *P* < 0.05), but the highest levels were observed when cells were cultured in both IL‐4 and IL‐25 (22‐fold, *P* < 0.05; Fig. 3A). As expected, exogenous IL‐4 increased the proportion of IL‐4‐expressing cells (11.2% ± 1.4 vs. 6.9% ± 1.0 for NP, *P* < 0.05; Fig. 3B). However, we found that addition of IL‐25 also increased the proportion of IL‐4^+^ cells (11.5% ± 1.3, *P* < 0.05), though the effect of adding both IL‐25 and IL‐4 appeared similar to either cytokine alone (14.2 ± 1.4%; Fig. 3B). There was also no difference in expression of IL‐4 message in cells cultured with exogenous IL‐4 (14.1‐fold) compared to IL‐25 (9.4‐fold; Fig. 3C). The proportion of GATA3^+^ cells was significantly higher when IL‐4 was added (19.4 ± 4.2% vs. 3.8 ± 0.8% for NP, *P* < 0.05) as well as when IL‐25 was added (7.7 ± 1.2% vs. NP, *P* < 0.05). In the presence of both IL‐25 and IL‐4, the % of GATA3^+^ cells was similar to that of IL‐4 alone (22.3 ± 3.4%; Fig. 3D). We also found that IL‐25 increased the proportion of CRTh2^+^ cells (2.9 ± 0.7% vs. 0.8 ± 0.3% for NP, *P* < 0.05), though the effect was lower than when IL‐4 was added (12.5 ± 3.2%, Fig. 3E). Addition of both IL‐4 and IL‐25 did not result in a significantly higher proportion of CRTh2^+^ cells (15.7 ± 3.8%) than IL‐4 alone (12.5 ± 3.2%). These data show that IL‐25 treatment induces IL‐4 expression from naïve CD4^+^ T cells to a similar extent as exogenous IL‐4. Furthermore, they show that the proportions of GATA3^+^ and CRTh2^+^ cells were significantly higher than control conditions, indicating IL‐25 can initiate Th2 differentiation, though not as well as IL‐4. We also assessed the influence of these conditions on cell growth. Figure 3F shows there were no effects of IL‐25 on cell growth at any time point, though cells cultured in both IL‐4 and IL‐25 showed a significant difference in absolute cell number at day 14. These data indicate that the Th2 differentiating effect of IL‐25 is likely not due to its influence on cell growth.

### The effect of IL‐25 on human Th2 cell differentiation is dependent on the IL‐4 pathway

**Figure 4:**
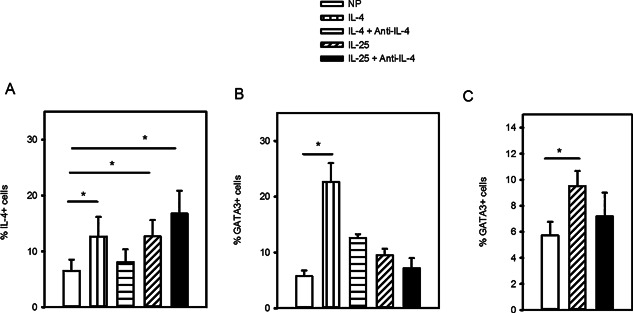
Effect of IL‐25 on GATA3 expression is dependent of IL‐4. Naïve CD4^+^ T cells were differentiated in non‐polarizing (NP) control conditions (αCD3/αCD28, IL‐2, αIFNγ and αIL‐12), NP + IL‐4 (IL‐4) or NP + IL‐25 (IL‐25) in the presence or absence of anti‐IL‐4. (A) Intracellular IL‐4, (B) GATA3 staining for all conditions and (C) GATA3 staining specifically for NP or IL‐25 or IL‐25 with anti‐IL‐4 (day 7,14, *n* = 5, three independently differentiated lines). Statistical significance determined by repeated measures ANOVA (A, B) and paired *t* test (C), *P < 0.05.

The effect of IL‐25 on Th2 differentiation of mouse CD4^+^ T cells has been reported to be through its ability to induce endogenous IL‐4 (38). To investigate whether this is also true in human T cells, we performed another set of differentiation experiments in the presence or absence of neutralizing antibody. These experiments replicated our previous finding (Fig. 3B), showing that IL‐25 induced a similar proportion of IL‐4^+^ cells (12.7 ± 2.9) compared to those treated with IL‐4 (12.6 ± 3.5, Fig. 4A). However, cells differentiated in IL‐4 conditions exhibited loss of % IL‐4^+^ cells in the presence of the IL‐4 neutralizing antibody (8.1 ± 2.9), while cells cultured with IL‐25 showed no drop in % IL‐4^+^ cells (16.7 ± 4.1%; Fig. 4A). We again found that the % GATA3^+^ cells were increased more by IL‐4 (22.6 ± 3.4) than by IL‐25 (9.52 ± 1.1%; Fig. 4B). The IL‐25 effect was significantly higher than NP (5.7 ± 1.0, *P* < 0.05; Fig. 4C) and this effect was lost in the presence of neutralizing IL‐4 antibody (7.2 ± 1.8, *P* > 0.05; Fig. 4B and C). These findings indicate that the influence of IL‐25 on naïve T cells involves activation of IL‐4 expression and that its effect on GATA3 appears to involve IL‐4.

### IL‐25 increases expression of IL‐5 and IL‐13 during Th2 differentiation

**Figure 5:**
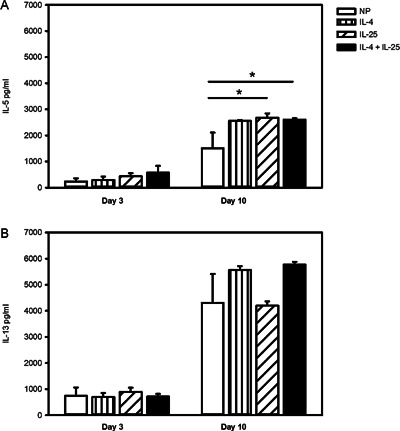
IL‐25 increases Th2 effector function. Naïve CD4^+^ T cells were differentiated in non‐polarizing (NP) control conditions (αCD3/αCD28, IL‐2, αIFNγ and αIL‐12), NP + IL‐4 (IL‐4), NP + IL‐25 (IL‐25) or NP + IL‐4 and IL‐25 (IL‐4+IL‐25). Supernatants were assayed for (A) IL‐5 or (B) IL‐13 production at day 3 (*n* = 4) and day 10 (*n* = 4; four independently differentiated cell lines). Statistical significance determined by repeated measures ANOVA, *P < 0.05.

IL‐25 has been shown to enhance expression of Th2 effector cytokines from both human memory Th2 cells 5 and ILC2 6, 7. Here we investigated whether IL‐25 could induce expression of IL‐5 and IL‐13 from naïve CD4^+^ T cells during Th2 differentiation. Figure 5A shows that when cells were examined after 3 days of differentiation there was no difference in IL‐5 levels across the various conditions (NP, 239.8 ± 119.7 pg/mL; IL‐4, 290.3 ± 139.6; IL‐25, 432.8 ± 121.9; IL‐4 + IL‐25, 575.0 ± 258.4). However, after 10 days of differentiation we observed that IL‐25 (2,681 ± 164.4) and IL‐4 + IL‐25 (2601 ± 60.15) significantly induced IL‐5 expression over NP (1513 ± 597.5, *P* < 0.05), while the effect of IL‐4 alone was not significant (2553 ± 38.50). Production of early IL‐13 (either day 3 or day 10) was not significantly different between conditions (Fig. 5B). When cells were examined after longer periods (day 17 to 38) of culture, in experiments examining the effect of adding IL‐25 to Th2 conditions (NP + IL‐4), we observed that differentiation with both IL‐25 and IL‐4 resulted in significantly more IL‐13 (60,564 ± 13,222 pg/mL), compared to cells cultured in IL‐4 (36,576 ± 7,399.5, *P* < 0.05; Table 1), though no effect of IL‐25 on IL‐5 expression was observed at these time points. Interestingly, the level of both IL‐5 and IL‐13 increased over the course of culture (Table 1).

**Table 1:** IL‐25 Increases Th2 effector function

	IL‐4	IL‐4 + IL‐25
	IL‐5 pg/ml	
Day 17 (*n* = 3)	15,430 ± 7,324.9	14,833 ± 335.85
Day 24 (*n* = 2)	49,107 ± 28,207^#^	33,798 ± 15,917^#^
Day 31/38 (*n* = 2)	59,763 ± 44,213^#,Ŧ^	66,355 ± 26,182^#,Ŧ^
Total (*n* = 7)	37,719 ± 11,745	34,972 ± 9,914.9
	IL‐13 pg/ml	
Day 17 (*n* = 3)	14,328 ± 9,717.0	10,889 ± 3,239.5
Day 24 (*n* = 3)	40,446 ± 10,697	56,427 ± 10,746
Day 31/38 (*n* = 4)	50,360 ± 10,742^¥^	100,923 ± 10,200^*,¥^
Total (*n* = 10)	36,576 ± 7,399.5	60,564 ± 13,322*

Data represent mean and SEM. When *n* < 3, SD was used^#^. Statistical significance between conditions determined by paired *t* test, *P < 0.05; Statistical significance for IL‐5 time points (day 17 *vs* day 24/31/38) determined by *t* test, ^Ŧ^ *P* < 0.05; Statistical significance for IL‐13 time points (day 17 *vs* day 24 *vs* day31/38) determined by ANOVA, ^¥^ *P* < 0.05.

### Regulation of the IL‐25R on differentiated CRTh2^+^ Th2 cells

**Figure 6:**
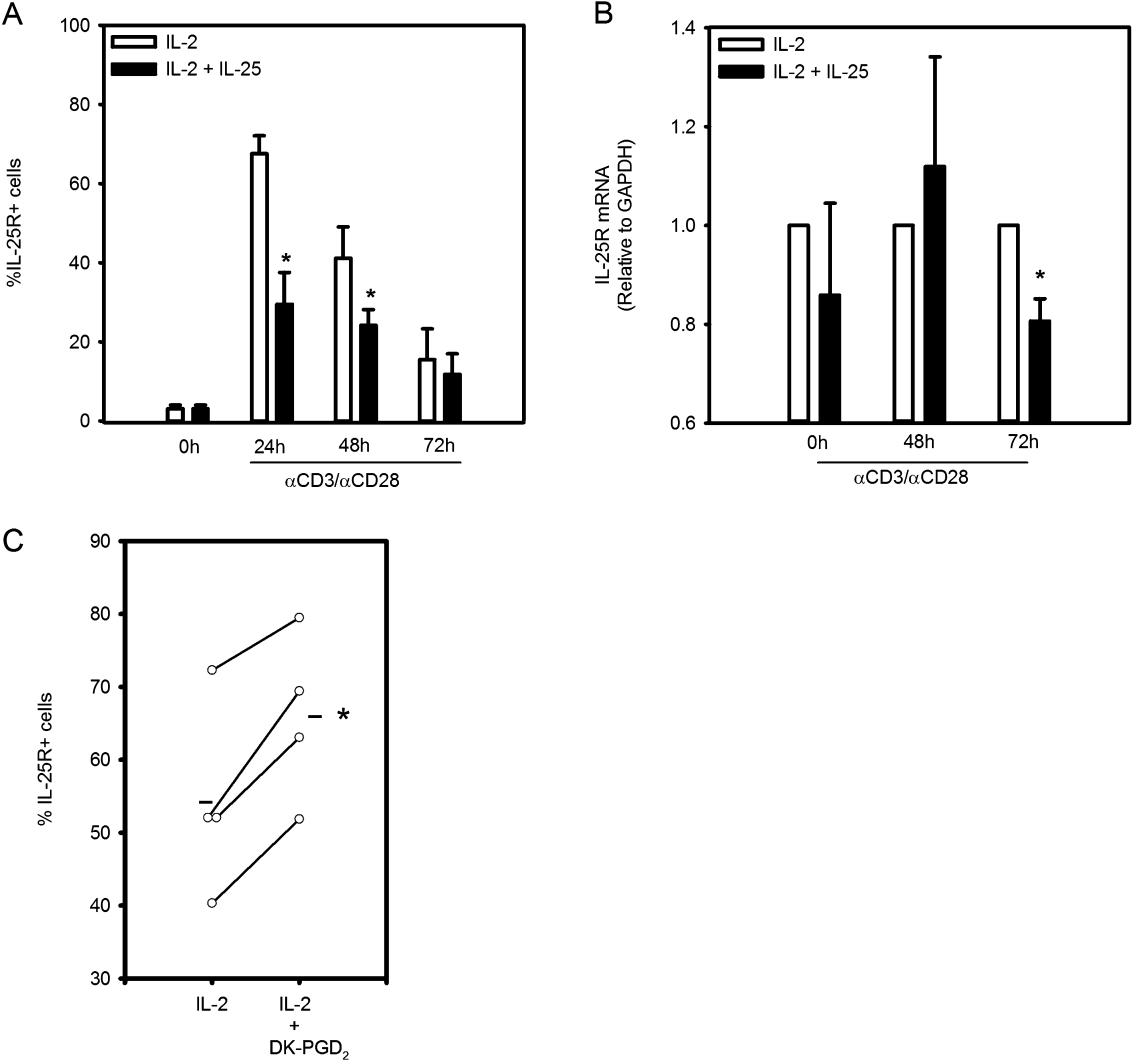
Regulation of IL‐25R on Th2 cells. Differentiated CRTh2^+^ Th2 cells were cultured with IL‐2 in the presence or absence of IL‐25 and stained for (A) IL‐25R (*n* = 5, three independently differentiated cell lines) or assessed for (B) IL‐25R mRNA levels (*n* = 3). (C) Surface expression of IL‐25R on CRTh2^+^ Th2 cells treated (24 h) with a CRTh2 agonist (DK‐PGD_2_) or IL‐2 (*n* = 4). Statistical significance determined by paired *t* test within each time point (A, B) or between conditions (C), *P < 0.05.

Regulation of IL‐25R expression on differentiated CRTh2^+^ Th2 cells has not been well characterized. We observed that surface IL‐25R expression is maximally induced after 24 h (>70%, Fig. 1E) and that IL‐25 induces expression of IL‐25R mRNA during differentiation (Fig. 3A). However, when we examined differentiated CRTh2^+^ Th2 cells we found that addition of IL‐25 to activated cells reduced surface receptor at 24 h (29.5 ± 8.1% vs. 67.5 ± 4.6% for IL‐2, *P* < 0.05) and 48 h (24.2 ± 4.0% vs. 41.2 ± 7.9%, *P* < 0.05; Fig. 6A), though IL‐25R mRNA levels were lower only after 3 days of activation (*P* < 0.05, Fig. 6B). CRTh2 is a receptor for PGD_2_ and activation through this pathway mediates Th2 cell function 33, 36. Therefore we also examined whether CRTh2 activation could enhance IL‐25R expression. In order to study this, surface IL‐25R was stained before and after exposure (24 h) to the CRTh2‐specific ligand, DK‐PGD_2_. Figure 6C shows that surface expression of IL‐25R expression was significantly increased in response to CRTh2 activation (66.0 ± 5.8% vs. 54.2 ± 6.6% for IL‐2, *P* < 0.05).

### IL‐25 slows re‐expression of CRTh2 following TCR activation

**Figure 7:**
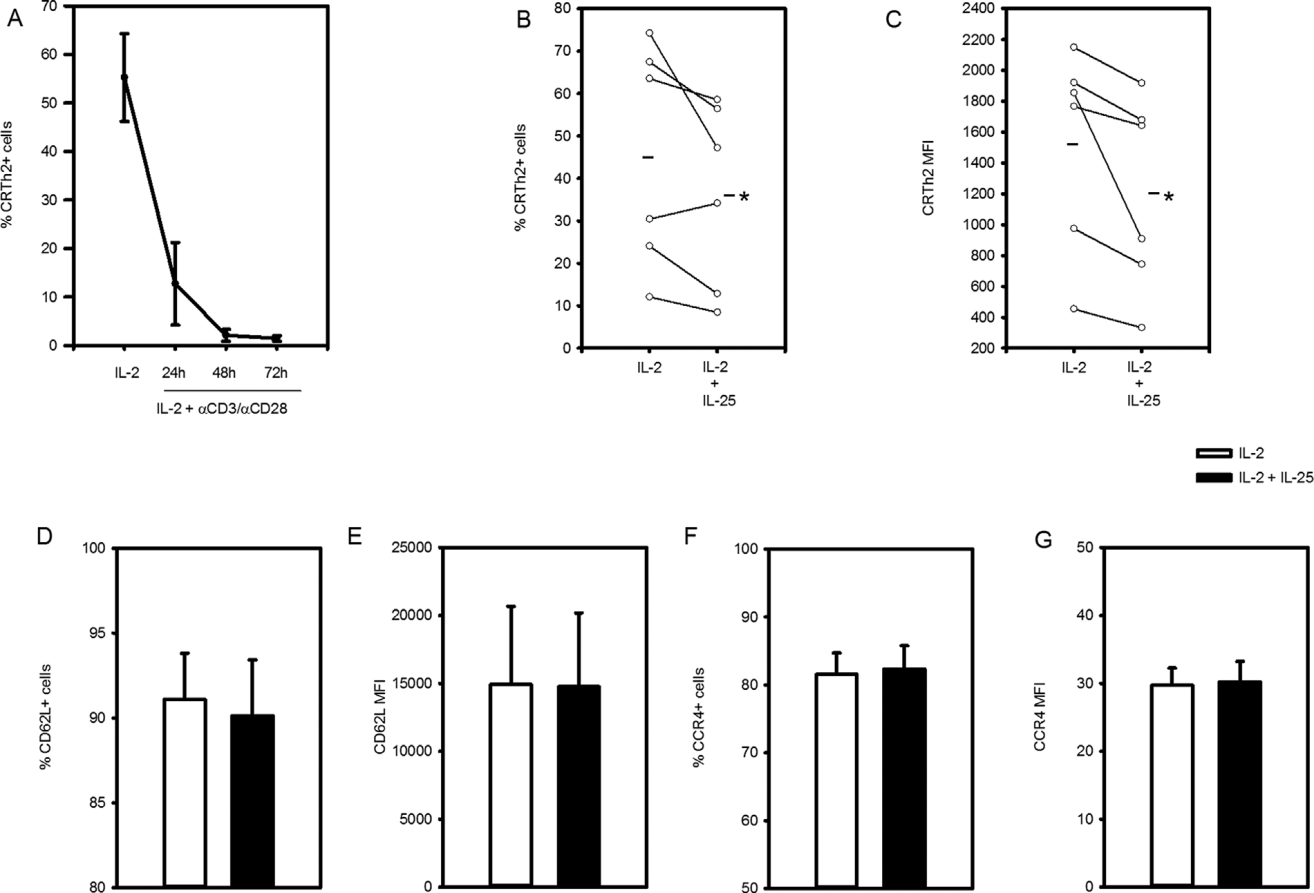
IL‐25 slows re‐expression of CRTh2 on Th2 cells following TCR activation. (A) Differentiated CRTh2^+^ Th2 cells cultured with IL‐2, αCD3 and αCD28 exhibited time dependent loss of surface CRTh2 expression (*n* = 4, two independently differentiated cell lines). (B–G) CRTh2^+^ Th2 cells were cultured with IL‐2, αCD3 and αCD28 (24 h) and then placed with IL‐2 in the presence or absence of IL‐25 (another 24 h). % of cells expressing (B) CRTh2, (D) CD62L or (F) CCR4 and mean fluorescent intensity (MFI) for (C) CRTh2, (E) CD62L and (G) CCR4 were quantified (*n* = 6, two independently differentiated lines). Statistical significance was determined by paired *t* test (B–G), *P < 0.05.

Since we observed that IL‐25 could induce CRTh2 expression by naïve T cells (Fig. 3), we investigated whether it enhances expression by differentiated CRTh2^+^ Th2 cells. Interestingly, while expression of the IL‐25R is highest after activation (Fig. 1E), CRTh2 expression is lowered by activation 3. Indeed, we observed a time dependent loss of surface CRTh2 expression following TCR crosslinking (Fig. 7A). To overcome this issue, cells were activated for 24 h (αCD3/αCD28 + IL‐2) to induce IL‐25R expression and then taken off stimulus and replated with IL‐2 or IL‐2 + IL‐25 for another 24 h. Fig. 7B shows that CRTh2 expression recovers somewhat in IL‐2 conditions (45.3 ± 10.7%) and is higher than cells treated with IL‐2 + IL‐25 (36.3 ± 8.8%, *P* < 0.05). Abundance of CRTh2 on a per cell basis, as determined by mean fluorescence intensity (MFI), was also lower when cells were cultured in IL‐2 + IL‐25 (1204 ± 257, vs. 1520 ± 268 for IL‐2, *P* < 0.05; Fig. 7C). IL‐25 did not, however, change expression of the central memory T cell marker, CD62L (94.6 ± 0.5% vs. 95.2 ± 0.5%, Fig. 7D/E) or the chemokine receptor CCR4 (82.3 ± 3.5% vs. 81.5 ± 3.2%; Fig. 7F/G). These data indicate that IL‐25 treatment has a negative impact on re‐expression of CRTh2 by differentiated Th2 cells when activated through the TCR.

## Discussion

A diverse range of environmental exposures can trigger innate immune responses that shape an individual's propensity for Th2 immunity (reviewed in 1, 43). Viruses 11, 44 and allergens can induce production of IL‐25 from airway epithelium and other inflammatory cells 5, 45. IL‐25 has been shown to promote Th2 cytokine expression from memory Th2 cells co‐stimulated with TSLP‐treated dendritic cells 5 as well as from ILC2 6, 7. Here we show that IL‐25 can act directly on naïve CD4^+^ T cells to initiate Th2 differentiation by inducing their expression of IL‐4, GATA3 and CRTh2. We also found that IL‐25 slows recovery of CRTh2 expression after T cell activation, indicating it may influence the resolution phase of inflammation.

A central tenet of Th2 differentiation is that IL‐4 promotes its own expression and responsiveness, as IL‐4 signaling induces IL‐4 receptor expression 46. We observed that IL‐25 also mediated expression of its own receptor from naïve CD4^+^ T cells, more potently than IL‐4, indicating that it exerts positive feedback during Th2 differentiation. Since IL‐4 induced surface IL‐25R, this strongly suggests that IL‐25 does as well, though we did not detect a difference in the level of surface IL‐25R across in the various culture conditions (NP vs. IL‐25, NP vs. IL‐4 or NP vs. IL‐4+IL‐25; data not shown). This was likely due to the staining being performed after resting (day 7 and 14), in an effort to compare expression with CRTh2 (which is downregulated by activation), rather than a time point designed to capture maximal IL‐25R expression (i.e. after activation). On the other hand, IL‐25 did not increase IL‐25R expression on fully differentiated CRTh2^+^ Th2 cells. In fact, we found that surface levels of IL‐25R were reduced after 24 h of IL‐25 treatment, though mRNA was lower only after 3 days. As such, IL‐25 binding to IL‐25R may result in receptor internalization and/or interfere with the epitope for the anti‐IL‐25R antibody. This issue may have also contributed to the difficulty in detecting surface IL‐25R in the IL‐25 differentiating cultures. IL‐25 did not appear to influence cell growth suggesting the early loss of surface expression may be due to receptor internalization. The ability of IL‐25 to increase IL‐25R expression during differentiation could indicate the presence of this cytokine influences an individual's Th2 cell responsiveness to future exposures that induce IL‐25. For instance, the IL‐25R mediates RSV‐induced Th2 inflammation and airway responses in mouse models 8 and so the level of IL‐25R may play a role in sensitivity to RSV, a risk factor for developing allergic asthma 47. Support for IL‐25 linking viral infection with allergic disease also comes from a study showing that airway levels of IL‐25 were elevated in asthmatics experimentally infected with rhinovirus 48. A recent report indicates that these exposures may contribute to asthma symptomatology, since IL‐25 levels were associated with low lung function, high serum IgE, sputum eosinophils and responsive to corticosteroid response 49.

Head‐to‐head comparison revealed that treatment with IL‐25 induced a similar proportion of IL‐4^+^ cells and level of IL‐4 mRNA as exogenous IL‐4, demonstrating that IL‐25 drives acquisition of IL‐4 expression. However, the fact that neutralizing antibody against IL‐4 reduced the proportion of IL‐4‐, but not IL‐25‐, induced cells indicates the IL‐25 effect is IL‐4‐independent, unlike in mice 38. Though IL‐25R signaling is not yet fully described, it activates NFκB 50, 51, a transcription factor that can upregulate IL‐4 21, and recently was shown to also signal through STAT5 52. STAT5 mediates IL‐2R signaling 27 and binds hypersensitive sites within the IL‐4 locus 53. Like the canonical Th2 transcription factor STAT6, STAT5 may also contribute to chromatin remodeling during Th2 differentiation and has been shown to be indispensable in the absence of STAT6 54. The fact that, exogenous IL‐4 induced more GATA3 than IL‐25, likely reflects a superior ability of the IL‐4‐STAT6 pathway to induce GATA3 22, 55. The IL‐25 effect on IL‐4 could also be due to its ability to induce expression of other Th2 transcription factors such as NFAT, JunB and cMAF 5, 38. IL‐4 −/− mice still develop some Th2 cells 56 and so a role for factors other than IL‐4 driving Th2 differentiation is considered likely. Our data suggest that, if present, IL‐25 could serve as a Th2 polarizing cytokine, albeit less potent than IL‐4. These findings are contrary to a recent report by Mearns et. al. who argue that IL‐25 is not a Th2 polarizing cytokine 57. This difference may be due to experimental design, as they used IL‐4 reporter mice which have a genomic insertion of GFP that disrupts an important IL‐4 regulatory element in the first intron. Therefore, if IL‐25 uses this locus or any others also perturbed by the insertion, the influence on IL‐4 production would not be observed.

The proportion of cells expressing GATA3 was higher in cells treated with IL‐25 compared to control and this effect was blocked by adding anti‐IL‐4. This suggests that a main influence of IL‐25 on Th2 differentiation is likely at the initiation phase, inducing IL‐4 that leads to the IL‐25‐mediated effects on GATA3 and CRTh2 expression. However, the reduction in % GATA3^+^ cells in the presence of antibody neutralizing IL‐4, but not a reduction in the % IL‐4^+^ cells, suggests that IL‐25 may also influence other aspects of the IL‐4 pathway. STAT5 mediates the IL‐2 increase in IL‐4Rα expression 53, in addition to influencing IL‐4 expression. Therefore, IL‐25R activation of STAT5 52 may enhance responsiveness of differentiating Th2 cells to IL‐4 by increasing expression of IL‐4Rα, thereby also participating in the reinforcement stage of Th2 differentiation 28.

The ability of IL‐25 to induce Th2 differentiation in the absence of a paracrine source of IL‐4 indicates its direct effect on CD4^+^ T cells may be another mechanism linking innate responses and development of Th2 immunity. Though differentiation is an event primarily considered to occur within the lymph nodes following dendritic cell migration, naïve CD4^+^ T cells are present at peripheral sites such as the lung 58 and therefore IL‐25 from epithelial cells 12 and/or eosinophils and basophils 5 could trigger local Th2 differentiation. However, TSLP‐treated dendritic cells can induce IL‐25R on Th2 memory cells 5 and IL‐25 has been shown to upregulate dendritic cell expression of Jagged 1 8, a Notch ligand and Th2 polarizing co‐stimulatory molecule 59. Thus, whether within the periphery or lymph nodes, the ability of IL‐25 to initiate Th2 differentiation through direct action on CD4^+^ T cells is likely enhanced *in vivo* by its ability to induce a Th2 favouring dendritic cell phenotype.

A caveat is that a small percentage of the starter cultures may have been memory T cells, since our staining showed they were only 93–95% CD45RA^+^. However, it is unlikely that our results were substantially influenced by inclusion of *in vivo* differentiated Th2 memory cells, as the proportion would be represented similarly in all experimental conditions and readily expanded in the non‐polarizing conditions (NP, containing αCD3/αCD28 and IL‐2). On the contrary, we observed only a low percentage of CRTh2^+^ cells, a marker of memory Th2 cells 3, in the NP condition (Fig. 3E, 0.8 ± 0.3%). As such, our data most likely reflect the influence of IL‐25 on Th2 differentiation, though we cannot rule out that some of the signal may be due to enhancing Th2 polarization of memory cells present at the start of culture.

Although IL‐25 did not increase IL‐25R on differentiated Th2 cells, activation through the TCR and stimulation with a CRTh2 agonist resulted in higher proportions of cells expressing IL‐25R. These data indicate Th2 cell responsiveness to IL‐25 is increased when cells encounter antigen and/or CRTh2 activation. Interestingly, IL‐25 slowed re‐expression of CRTh2 on differentiated Th2 cells following TCR activation, though we did not observe a change in surface expression of the memory T cell marker CD62L or CCR4, a chemokine receptor also considered a marker of the Th2 cell phenotype 60. Activation through CRTh2 has been shown to mediate movement of memory CD4^+^ T cell across lymphatic vessel endothelial cells *in vitro*, a model used to study how cells exit the periphery to recirculate back to the lymph nodes during the resolution phase 37. Therefore our findings suggest that, in addition to mediating Th2 effector cytokine production, IL‐25 may also play a role in retaining Th2 cells within tissues, interfering with clearance of inflammation.

In summary, IL‐25 has been shown to play a role in Th2 responses and models of allergic asthma 10, 61, 62. We demonstrate here that these effects may, at least partially, be through direct action of IL‐25 on CD4^+^ T cells and differentiated CRTh2^+^ Th2 cells. IL‐25 also induced expression of its own receptor, indicating it may enhance sensitivity to environmental stimuli that trigger IL‐25 production.

## Conflicts of Interest

The authors have no conflicts of interest as all funds were awarded by peer‐reviewed government run granting agencies.
